# Signs and symptoms do not predict, but may help rule out acute Q fever in favour of other respiratory tract infections, and reduce antibiotics overuse in primary care

**DOI:** 10.1186/s12879-020-05400-0

**Published:** 2020-09-21

**Authors:** Volker H. Hackert, Nicole H. T. M. Dukers-Muijrers, Christian J. P. A. Hoebe

**Affiliations:** 1 Department of Sexual Health, Infectious Diseases, and Environmental Health, South Limburg Public Health Service, Heerlen, 6411 TE The Netherlands; 2 Department of Social Medicine and Medical Microbiology, Care and Public Health Research Institute (CAPHRI), Faculty of Health, Medicine and Life Sciences, Maastricht University / MUMC+, Maastricht, 6229 HX The Netherlands

**Keywords:** Q fever, *Coxiella burn*e*tii*, Respiratory tract infection, Prediction, Antibiotics, Primary care

## Abstract

**Background:**

From early 2009, the Dutch region of South Limburg experienced a massive outbreak of Q fever, overlapping with the influenza A(H1N1)pdm09 pandemic during the second half of the year and affecting approximately 2.9% of a 300,000 population. Acute Q fever shares clinical features with other respiratory conditions. Most symptomatic acute infections are characterized by mild symptoms, or an isolated febrile syndrome. Pneumonia was present in a majority of hospitalized patients during the Dutch 2007–2010 Q fever epidemic. Early empiric doxycycline, guided by signs and symptoms and patient history, should not be delayed awaiting laboratory confirmation, as it may shorten disease and prevent progression to focalized persistent Q fever. We assessed signs’ and symptoms’ association with acute Q fever to guide early empiric treatment in primary care patients.

**Methods:**

In response to the outbreak, regional primary care physicians and hospital-based medical specialists tested a total of 1218 subjects for Q fever. Testing activity was bimodal, a first “wave” lasting from March to December 2009, followed by a second “wave” which lasted into 2010 and coincided with peak pandemic influenza activity. We approached all 253 notified acute Q fever cases and a random sample of 457 Q fever negative individuals for signs and symptoms of disease. Using data from 140/229(61.1%) Q fever positive and 194/391(49.6%) Q fever negative respondents from wave 1, we built symptom-based models predictive of Q-fever outcome, validated against subsets of data from wave 1 and wave 2.

**Results:**

Our models had poor to moderate AUC scores (0.68 to 0.72%), with low positive (4.6–8.3%), but high negative predictive values (91.7–99.5%). Male sex, fever, and pneumonia were strong positive predictors, while cough was a strong negative predictor of acute Q fever in these models.

**Conclusion:**

Whereas signs and symptoms of disease do not appear to predict acute Q fever, they may help rule it out in favour of other respiratory conditions, prompting a delayed or non-prescribing approach instead of early empiric doxycycline in primary care patients with non-severe presentations. Signs and symptoms thus may help reduce the overuse of antibiotics in primary care during and following outbreaks of Q fever.

## Background

From March 2009, South Limburg, the southernmost region of the Netherlands, experienced a massive outbreak of human Q fever related to an abortion storm on a local dairy-goat farm. Laboratory-confirmed symptomatic human Q-fever cases were first reported in April, peaked in May, and then steadily declined over subsequent months. Culling of infected goats took place around the turn of the year. By April 2010, no more new cases were reported to the regional Public Health Service (PHS), and the number of notified human cases reported to the regional PHS had totalled 253, whereas the number of infections was estimated to run into thousands [1].

A majority of acute Q fever infections are understood to be asymptomatic or only mildly symptomatic. Symptomatic patients usually present with a febrile syndrome or flu-like illness frequently said to be associated with myalgia and headache. During the Dutch Q fever epidemic, which lasted from 2007 to 2010, pneumonia was present in as many as 86% of hospitalized patients. Although most cases of acute Q fever are self-limiting, early antibiotic treatment with doxycycline within the first days of symptoms may shorten duration of disease, and may prevent progression to persistent focalized infection, commonly referred to as chronic Q fever, in cases with underlying risk factors, including vascular and valvular anomalies [2, 3]. In patients with known valvular heart disease, combining doxycycline with hydroxychloroquine has been shown to prevent progression to Q fever endocarditis [4, 5]. Definitive diagnosis usually relies on laboratory testing. While polymerase chain reaction (PCR) may provide timely outcomes, serological assays still are the mainstay of laboratory testing, resulting in diagnostic delay and foregone or inappropriate treatment [2].

During the Dutch epidemic of Q fever, general practitioners (GP’s) with experience in treating Q fever patients tended to start empiric antibiotic therapy ahead of laboratory confirmation, which had a median delay of 20 days from onset of illness in 2009 [6, 7]. While doxycycline was the most commonly prescribed initial antibiotic, a substantial proportion of subjects were treated with a penicillin, which is considered to be ineffective in Q fever [8]. A complicating factor in the diagnostic workup of cases was the influenza A(H1N1)pdm09 pandemic which overlapped with the regional outbreak for several months during the second half of the year.

Several studies have assessed the diagnostic potential of signs and symptoms in respiratory disease, including influenza and Q fever [9–12]. However, evidence regarding the predictive usefulness of signs and symptoms in patients with suspected Q fever is scarce, and has been limited to hospital settings. A Dutch study performed during the 2007–2010 Q fever epidemic in the Netherlands, for example, found that signs and symptoms did not differentiate between acute Q fever and other respiratory infections in hospitalized patients [13]. However, it is the primary care setting where signs and symptoms of disease are essential in the initial diagnostic workup and in guiding early clinical decision-making. Our study, which used data from a cohort of subjects a majority of whom were tested by general practitioners, aimed to assess whether signs and symptoms could support decision-making in primary care. Specifically, we assessed whether signs and symptoms could accurately identify acute Q fever in suspect cases prior to laboratory confirmation, or help rule out the diagnosis in favour of other respiratory infections where, depending on national guidelines, treatment with amoxicillin as a first-line antibiotic or a delayed or non-prescribing approach would be considered more appropriate.

## Methods

### Study area

The study area was the catchment area of one of the largest Dutch general hospitals, located in South Limburg, Netherlands (346 km^2^, 12 municipalities, 308,000 inhabitants).

### Study period

In March 2009, the regional Food and Consumer Product Safety Authority notified the South Limburg PHS of a large dairy-goat farm where 220 out of 450 pregnant goats had aborted due to laboratory-confirmed Q fever. The study period was defined by the time of veterinary notification (March 2009) and the time when the outbreak source had been eliminated through culling of infected goats and vaccination of remaining goat populations, and new community cases were no longer reported (April 2010).

### Study design

We performed a retrospective case-control study assessing the association of acute Q fever case status with signs and symptoms of disease in a sample of questionnaire respondents from the cohort of all individuals tested for acute Q fever by GP’s or hospital-based medical specialists in the period from March 2009 through April 2010 (*n* = 1218). Medical specialists requesting tests were from a variety of fields, including internal medicine, infectious disease, or respiratory medicine. All notifiable community cases (*n* = 253) were reported to the regional PHS by the affiliated regional testing laboratory. Disease onset in community cases was physician-reported. The testing laboratory also provided data on all 965 non-notifiable Q fever negative individuals tested in the study period, including date of birth, gender, zip code as a proxy for residential address, name and address of GP, testing dates, and testing results. Promptly following notification, all notified community cases were approached with a questionnaire assessing the presence or absence of individual presenting signs and symptoms of disease preceding testing, underlying medical conditions, and risk exposure activities, among others. Response in this group was 64.4%(163/253). Among the 965 subjects who had tested negative (non-notifiable controls), a random selection of 457 individuals were approached with the same questionnaire via their GP’s (response: 67.2%(307/457)).

### Laboratory investigation

The entire cohort of subjects was tested for IgG- and IgM-type antibodies to phase-I and phase-II *C. burnetii* antigen by Serion ELISA classic, according to manufacturer’s instructions (Serion ELISA classic, Institut Virion\Serion GmbH, Würzburg, Germany). ELISA-positive specimens were subjected to confirmation by indirect immunofluorescent antibody assay (IFA) (*C. burnetii* IFA IgM/IgG Test Kit, Fuller Laboratories, Fullerton, California). PCR was routinely performed on all ELISA-negative samples. The presence of phase-II IgM antibodies to *C. burnetii* (absorbance > 10% above extinction of the cut-off control) in a single serum sample, confirmed by IFA, or the presence of *C. burnetii* DNA in PCR (cycle threshold ≤36) was considered diagnostic of acute Q fever [14–16].

### Study population

**Fig. 1:**
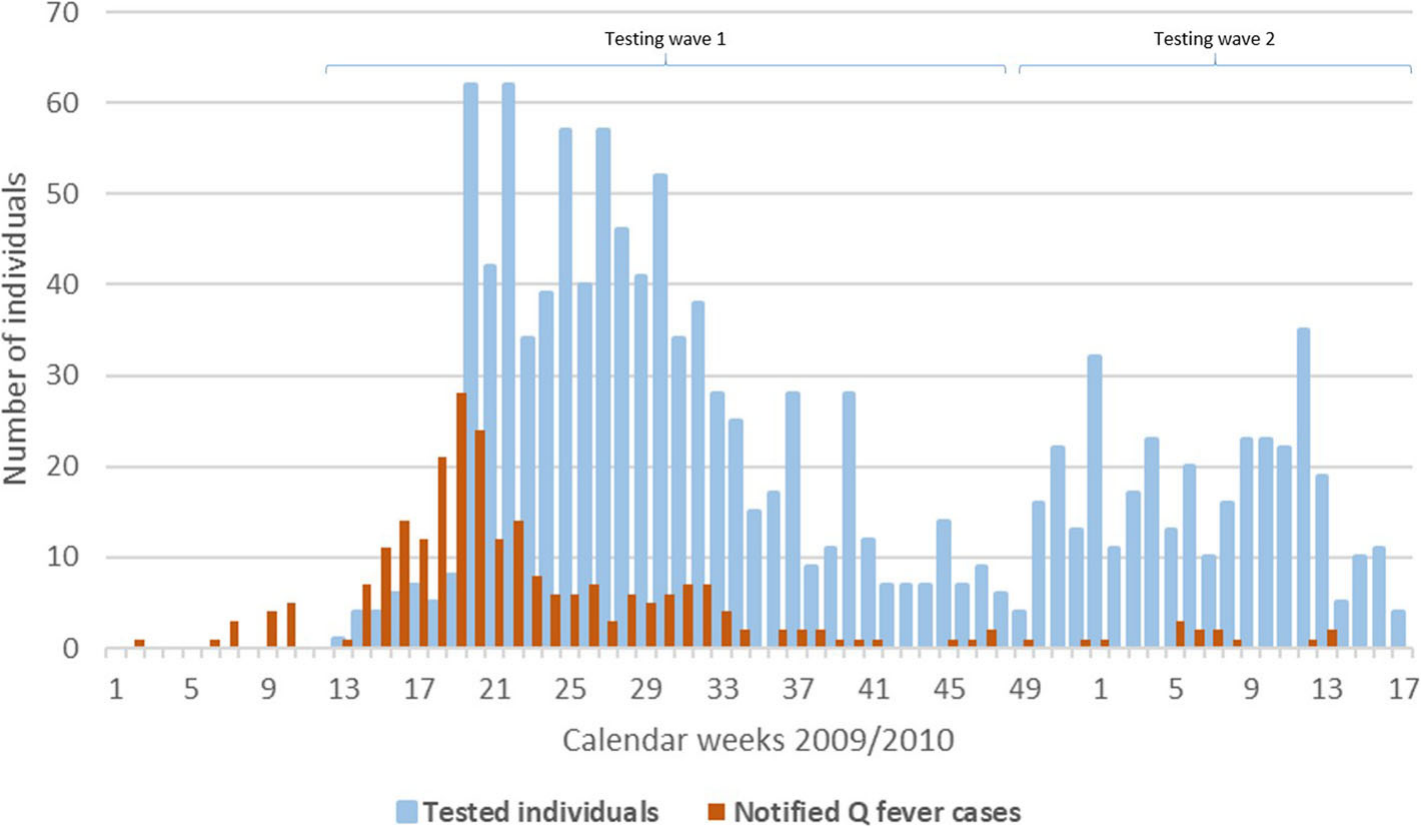
Weekly counts of all individuals tested for Q-fever by South Limburg GP’s and hospital-based medical specialists (*n* = 1218), along with weekly counts of notified Q-fever cases (by GP-reported week of disease onset, *n* = 253)

Overall, 20.8% (253/1218) of all patients tested were confirmed with a diagnosis of acute Q fever by serology or PCR. Testing activity followed a bimodal distribution over time. A larger first testing wave from March to December 2009 (wave 1) was followed by a second smaller one from December 2009 through April 2010 (wave 2) (Fig. 1). The larger first wave, including subjects tested from week 13 (March 2009) until week 49 (December 2009), contained 72% of all tested patients, with a Q fever positive rate of 26%, thus yielding 91% of all notifiable patients with a laboratory-confirmed diagnosis of acute Q fever in the study period. By contrast, the second wave, although it counted more than a quarter of all tested patients, had a positive rate of only 7% and identified just 9% of all notified patients. Characteristics of tested subjects are summarized in Table 1.

**Table 1:** Characteristics of the total population of subjects tested in the study period, by testing wave and testing outcome for acute Q fever (*n* = 1218)

	Q fever positive		Q fever negative		Total	Positive rate
**Wave 1**						
Subjects tested, n	229		644		873	26.2%
Test ordered by						
GP, n (%)	155	(67.7)	468	(72.2)		
Medical specialist, n (%)	72	(31.4)	170	(26.4)		
Unknown, n (%)	2	(0.9)	6	(0.9)		
Age in years (week 13, 2009)						
Mean (range)	49.0	(0.9–85.5)	45.1	(0.5–92.4)		
0–19, n (%)	11	(4.8)	87	(13.5)		
20–39, n (%)	46	(24.9)	154	(37.4)		
40–59, n (%)	113	(49.3)	235	(36.5)		
≥60, n (%)	59	(25.8)	168	26.1)		
Sex, n female (%)	83	(36.2)	346	(53.7)		
Residential farm distance, mean km	5.1		6.1			
**Wave 2**						
Subjects tested, n	24		321		345	7.0%
Test ordered by						
GP, n (%)	19	(79.2)	237	(79.2)		
Medical specialist, n (%)			61	(19.0)		
Unknown, n (%)	5	(20.8)	23	(7.2)		
Age in years (week 13, 2009) )						
Mean (range)	46.7	(20.8–71.7)	46.8	(0–88.5)		
0–19, n (%)	0	0	42	(13.1)		
20–39, n (%)	4	(16.7)	60	(18.7)		
40–59, n (%)	17	(70.8)	125	(38.9)		
≥60, n (%)	3	(12.5)	94	(29.3)		
Sex, n female (%)	10	(41.7)	184	(57.3)		
Residential farm distance, mean km	5.1		5.7			

### Data collection and analysis

Statistical analyses were performed using SPSS Statistics 21.0 (IBM corporation, New York, USA). For derivation and validation of our symptom-based prediction, we used questionnaire data from all adult questionnaire recipients with a complete questionnaire response tested during wave 1, i.e., all questionnaire recipients from the age of 20 years who had been tested in the weeks before week 49 and had answered all questions about presenting signs and symptoms of disease which preceded testing. Of all 873 patients tested during wave 1, 620 (71.0%) had received the questionnaire, with response from 341 questionnaire recipients (response rate 55.0%), and a complete response from 334 recipients (complete response rate 53.9%). Children and adolescents under the age of 20 were excluded since the association of signs and symptoms with Q fever in this age group are known to be less clear-cut than in adults [17, 18]. A holdout sample of all subjects tested during wave 2 (i.e., the immediate post-outbreak phase) with a complete questionnaire response was used for additional validation of the models derived from our wave 1 data. Of all 345 patients tested during wave 2, 119 (34.5%) had received the questionnaire, with response from 113 questionnaire recipients (response rate 95.0%), and complete response from 109 recipients (complete response rate 91.6%). Characteristics of questionnaire respondents are summarized in Table 2.

**Table 2:** Characteristics of questionnaire respondents with complete questionnaire response, by testing wave and testing outcome for acute Q fever (*n* = 453)

	Q fever positive		Q fever negative	
**Wave 1**				
Questionnaire recipients, n (% of tested)	229	(100)	391	(60.7)
Respondents, n (response rate)	143	(62.4)	198	(50.6)
Respondents, complete response, n (rate)	140	(97.9)	194	(98.0)
Test ordered by				
GP, n (%)	102	(72.9)	177	(91.2)
Medical specialist, n (%)	38	(27.1)	16	(8.2)
Unknown, n (%)	0	(0)	1	(0.5)
Age in years (week 13, 2009)				
Mean (range)	49.7	(5.9–85.5)	46.7	(0.8–92.4)
0–19, n (%)	7	(5.0)	28	(14.4)
20–39, n (%)	23	(16.4)	40	(20.6)
40–59, n (%)	72	(51.4)	64	(33.0)
≥60, n (%)	38	(27.1)	62	(32.0)
Sex, n female (%)	51	(36.4)	106	(54.6)
Residential farm distance, mean km (range)	4.7	(1.9–13.1)	4.8	(1.1–11.6)
Active smoking, n (%)	49	(35.0)	47	(24.2)
**Wave 2**				
Questionnaire recipients, n (% of tested)	24	(100)	95	(29.6)
Respondents, n (response rate)	19	(79.2)	94	(98.9)
Respondents, complete response, n (rate)	18	(94.7)	91	(96.8)
Test ordered by				
GP, n (%)	13	(72.2)	73	(80.2)
Medical specialist, n (%)				
Unknown, n (%)	5	(26.3)	18	(19.8)
Age in years (week 13, 2009)				
Mean (range)	47.7	(20.8–71.7)	49.1	(4.4–82.3)
0–19, n (%)	0	0	9	(9.9)
20–39, n (%)	3	(16.7))	11	(12.1)
40–59, n (%)	12	(66.7)	43	(47.3)
≥60, n (%)	3	(16.7)	28	(30.8)
Sex, n female (%)	8	(44.4)	56	(61.5)
Residential farm distance, mean km (range)	4.3	(1.6–7.4)	5.1	(1.2–13.1)
Active smoking, n (%)	4	(22.2)	17	(18.7)

### Selection of predictors to be included in our prediction models

We first assessed association of Q fever status with sex, age, smoking habits, test ordered by GP versus (hospital-based) medical specialist, and presence or absence of individual presenting signs and symptoms of disease in all 334 complete questionnaire respondents tested during wave 1, using univariable logistic regression. For a full list of signs and symptoms assessed by our questionnaire refer to Table 3. As a next step, we assessed associations with Q fever status, entering the full set of variables into stepwise backward multivariable logistic regression, a procedure that eliminates statistically non-significant variables along the way. Variables that were statistically significantly (*p* < 0.10) associated with Q fever outcome in univariable or multivariable regression were selected for inclusion in our prediction models (refer to next paragraph). Sex, age, active smoking habits, and test ordered by GP versus medical specialist were selected as potential predictors regardless of their association with outcome in univariable or multivariable regression in the steps described above. Distance of residential address from the outbreak farm was not included as a candidate predictor, since this information would usually be unavailable to physicians at the time when patients present to their office, or may be unknown altogether in situations where no outbreak source has (yet) been identified.

**Table 3:** Univariable associations of acute Q fever outcome with potential predictors in adult respondents with complete questionnaire response tested during wave 1, *n* = 334 (Q fever positive *n* = 140, Q fever negative *n* = 194)

	B	S.E.	P	OR	95% CI	
					Lower	Upper
**Signs and symptoms**						
Fever	1.50	0.31	<0.001	4.47	2.43	8.23
Pneumonia	1.42	0.30	<0.001	4.12	2.29	7.41
Confusion	0.85	0.37	0.023	2.33	1.13	4.83
Flu-like illness	0.78	0.28	0.005	2.19	1.27	3.79
Night sweats	0.76	0.25	0.002	2.14	1.31	3.48
Weight loss	0.72	0.27	0.007	2.05	1.22	3.45
Severe fatigue	0.69	0.25	0.006	1.99	1.22	3.24
Headache	0.52	0.24	0.03	1.67	1.05	2.68
Chest pain	0.51	0.26	0.05	1.67	1.00	2.77
Abdominal pain	−0.81	0.28	0.004	0.45	0.26	0.78
Shortness of breath	0.40	0.25	0.10	1.49	0.92	2.41
Arthralgia	0.33	0.23	0.17	1.39	0.88	2.19
Stiff neck	0.12	0.24	0.62	1.12	0.71	1.79
Exanthema	0.06	0.28	0.84	1.06	0.62	1.82
Diarrhea	0.00	0.25	0.99	1.00	0.62	1.63
Jaundice	−0.65	0.70	0.36	0.52	0.13	2.07
Earache	− 0.51	0.34	0.14	0.60	0.31	1.17
Ocular symptoms	−0.36	0.40	0.37	0.70	0.32	1.52
Cough	−0.31	0.24	0.20	0.74	0.46	1.17
Myalgia	−0.06	0.23	0.79	0.94	0.59	1.48
**Demographics**						
Age 40–59 vs. 20–39	0.67	0.31	0.03	1.96	1.06	3.61
Age ≥60 vs. 20–39	0.06	0.33	0.85	1.07	0.56	2.05
Sex	0.83	0.24	0.001	2.30	1.43	3.67
Residential farm distance	−0.02	0.05	0.68	0.98	0.90	1.08
**Others**						
Active smoking	0.42	0.25	0.10	1.52	0.93	2.48
Medical specialist vs. GP	1.35	0.34	<0.001	3.85	1.97	7.53

### Prediction model derivation

The entire dataset of 334 complete questionnaire respondents tested during wave 1 was randomly split into four subsets, each including roughly 25% of respondents. One subset was set aside for validation (henceforth referred to as the validation subset), while data of the remaining three subsets combined (including roughly 75% of the 334 respondents, henceforth referred to as the prediction subset) were used for derivation of our prediction model. To build the prediction model, we used the prediction subset, entering all variables selected according to the procedure described in the previous paragraph into backward stepwise logistic regression. Coefficients obtained from the variables that were statistically significantly associated with Q fever outcome (*p* < 0.10) were used to calculate a sum score. Predictive performance of the model was then assessed by applying the score to the validation subset to determine Area Under the Curve (AUC) of the Receiver Operator Curve (ROC), sensitivity and specificity (based on cut-points specific to the model, calculated according to the Youden index), and the model’s positive (PPV) and negative predictive value (NPV) (based on an estimated regional seroprevalence of 2.9%, derived from comparison of two regional population samples, one pre-outbreak dating from 2008, and the second one post-outbreak dating from 2010) [1, 19]. For additional validation, the same score was applied to the immediate post-outbreak holdout sample from wave 2, again using AUC to assess predictive performance of the model. The entire process was repeated for the remaining three subsets, resulting in four prediction models, each applied once to its specific validation subset from wave 1, and once to the holdout sample from wave 2. Finally, we compared models in terms of their AUC’s of the ROC, assessing statistical differences between AUC’s using a bivariate approach [20, 21].

## Results

### Uni- and multivariable associations of acute Q fever outcome with potential predictors

Univariable associations of Q fever status with sex, age, smoking habits, test ordered by GP versus (hospital-based) medical specialist, and presence or absence of individual presenting signs and symptoms of disease in all 334 complete questionnaire respondents (Q fever positive *n* = 140, Q fever negative *n* = 194) tested during wave 1, based on univariable logistic regression, are summarized in Table 3. Statistically significant multivariable associations for the same set of 334 complete questionnaire respondents are summarized in Table 4, eliminating non-significant associations through backward stepwise logistic regression.

**Table 4:** Multivariable associations of acute Q fever outcome with potential predictors in adult respondents with complete questionnaire response tested during wave 1, *n* = 334 (Q fever positive *n* = 140, Q fever negative *n* = 194)

	B	S.E.	P	OR	95%CI	
Sex	0.56	0.32	0.083	1.75	0.93	3.30
Age 20–39 vs. 40–59	0.82	0.33	0.012	2.26	1.20	4.27
Fever	1.56	0.41	<0.001	4.77	2.15	10.59
Pneumonia	1.25	0.41	0.002	3.47	1.57	7.69
Confusion	1.14	0.56	0.04	3.12	1.04	9.32
Abdominal pain	−0.75	0.38	0.05	0.47	0.22	1.00
Cough	−0.62	0.35	0.08	0.54	0.27	1.07
Earache	−0.99	0.47	0.04	0.37	0.15	0.93
Constant	−1.80	0.46	<0.001	n.a.		

### Prediction models derived from the four prediction subsets (model 1 through 4)

Table 5 shows sets of statistically significant predictors, referred to as model 1 through 4, derived from backward stepwise logistic regression for the four prediction subsets including roughly 75% of the 334 respondents each. Coefficient, *P* value, standard error (SE), and odds ratio (OR) are included for each predictor, in addition to relevant model statistics.

**Table 5:** Multivariable prediction models derived from backward stepwise logistic regression on the four prediction subsets among 334 complete questionnaire respondents (model 1 through 4)

	Model 1				Model 2				Model 3				Model 4			
	B	P	SE	OR	B	P	SE	OR	B	P	SE	OR	B	P	SE	OR
**Variable**																
Sex	1.13	<0.001	0.33	3.09	0.66	0.04	0.32	1.94	0.56	0.08	0.32	1.75	0.91	0.01	0.32	2.48
Age 40–59 yrs					0.83	0.01	0.31	2.29								
Age ≥60 yrs	−0.91	0.01	0.37	0.40					0.82	0.01	0.33	2.26	−0.57	0.10	0.34	0.57
Active smoking					0.77	0.03	0.35	2.16	−0.62	0.08	0.35	0.54	0.87	0.01	0.34	2.39
Fever	0.83	0.07	0.46	2.30	1.24	<0.001	0.38	3.45	1.56	<0.001	0.41	4.77	1.25	<0.001	0.38	3.48
Flu-like illness	0.98	0.02	0.41	2.66												
Pneumonia	1.24	0.00	0.38	3.45	1.02	0.01	0.40	2.78	1.25	0.00	0.41	3.47	1.43	0.00	0.42	4.16
Confusion	0.99	0.06	0.53	2.68												
Severe fatigue	0.59	0.10	0.36	1.81												
Abdominal pain	−1.15	0.01	0.42	0.32	−0.76	0.05	0.38	0.47	−0.75	0.05	0.38	0.47	−0.88	0.02	0.37	0.42
Earache	−1.02	0.04	0.49	0.36												
Chest pain					0.61	0.08	0.35	1.84								
Cough					−0.63	0.05	0.32	0.53	−0.99	0.04	0.47	0.37	−0.86	0.01	0.33	0.42
Headache									1.14	0.04	0.56	3.12	0.64	0.06	0.35	1.90
Constant	−2.42	<0.001	0.56	n.a.	−2.15	<0.001	0.47	n.a.	−1.80	<0.001	0.46	n.a.	−1.55	<0.001	0.50	n.a.
χ^2^			71.00				52.00				65.00				67.00	
df			9.00				8.00				8.00				8.00	
P			<0.001				<0.001				<0.001				<0.001	
Nagelkerke R^2^			0.37				0.28				0.34				0.33	
Hosmer & Lemeshow			0.69				0.46				0.81				0.71	
Classification accuracy			75.1%				71.8%				73.1%				73.1%	

### Predictive performance of model 1 through 4

**Fig. 2:**
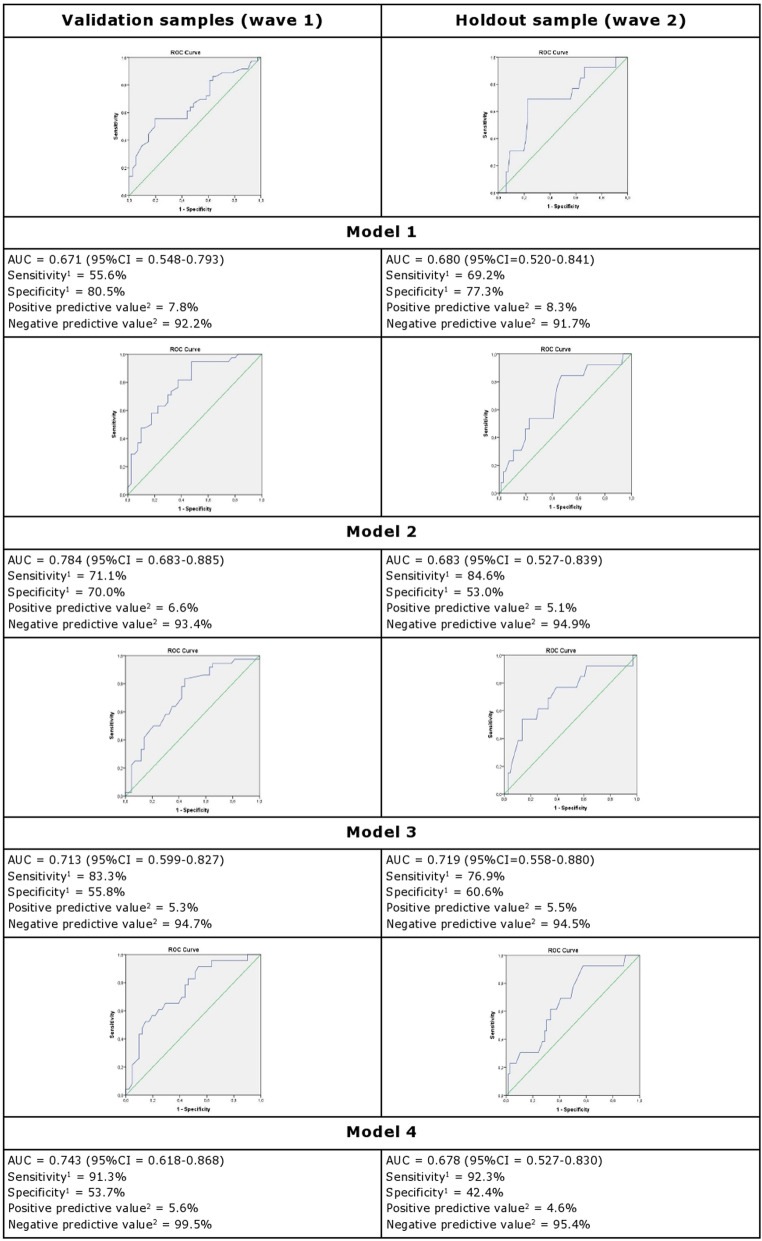
Predictive performance of the four prediction models tested on their corresponding validation subsets from wave 1 (left column) and the holdout sample from wave 2 (right column). (legend). ^1^at cut-point calculated according to Youden index ^2^based on cut-point calculated according to Youden index and an estimated regional prevalence of 2.9%

Figure 2 summarizes performance characteristics of the four prediction models, based on each model’s coefficient score applied to the corresponding validation subset from wave 1 (left column), and to the holdout sample from wave 2 (right column). AUC’s ranged from 0.671 to 0.784 from least to best performing model, generally considered to be poor to moderate in terms of predictive accuracy. Sensitivity of the models ranged between 55.6 and 92.3%, with specificities between 42.4 and 80.5%, PPV’s between 4.6 and 8.3%, and NPV’s between 91.7 and 99.5%.

The difference between model performance in terms of AUC was statistically significant between the least- and best-performing model applied to their corresponding validation subsets (model 1 versus model 2, *P* = 0.02), but not between the least- and best-performing model applied to the holdout sample (model 4 versus model 3, *P* = 0.73). Comparing performance of each model on the validation subset versus the holdout sample (rows in Fig. 2) showed no statistically significant differences either.

## Discussion

Given the poor to moderate performance of our prediction models, our study suggests that signs and symptoms of disease do not accurately predict acute human Q fever in GP patients, confirming findings from a Dutch study in hospitalized patients [13]. However, signs and symptoms may be useful in ruling out acute Q fever in favour of other acute lower respiratory tract infections. This is especially relevant in cases where pneumonia is not suspected and a non-prescribing or delayed prescribing approach would seem more appropriate, helping reduce the overuse of antibiotics. In the cohort of patients tested in our region, this would have been particularly relevant in the immediate post-outbreak phase where the number of tests for acute human Q fever remained high but the proportion of seropositive cases was very low (7%), and prevalence of pneumonia was also low (12%). Even during the outbreak phase, only 26.2% of tested individuals were Q fever positive, and ruling out acute Q fever by symptoms would likely have contributed to a reduction in antibiotic overuse.

Male sex, fever, and pneumonia were positive predictors of acute Q fever across all four of our models, in accordance with other studies [2, 22]. Cough was a negative predictor in three models, suggesting that cough as a symptom may be useful in ruling out Q fever in suspected cases. Cough is considered a common symptom in upper respiratory tract infections. Its presence, according to our findings, may point to respiratory conditions other than Q fever [23]. Specifically, cough has been described as a symptom suggestive of influenza, rather than, for example, common cold [9]. Overall, in our sample cough was the most prevalent symptom – second only to flu-like illness – in questionnaire respondents from the second wave, both in Q fever positive and Q fever negative subjects. This, combined with the low rate of Q fever positive findings during the second wave, may suggest that the rise in Q fever testing activity by GP’s and medical specialists during the second half of 2009 and the early months of 2010 may – at least to some degree – have resulted from patients presenting with respiratory symptoms due to increasing pandemic influenza A(H1N1)pdm09 activity in that period. Moreover, due to long persistence of anti-Coxiella phase II IgM following infection, some of the subjects who tested positive during the second wave may have been misclassified as acute Q fever. While abdominal pain was a negative predictor of acute Q fever across all four models, gastrointestinal symptoms such as abdominal pain and diarrhoea were much less prevalent than cough in both Q fever positive and Q fever negative subjects from both waves, and the nature of the observed negative association of abdominal pain with Q fever remains unclear. Studies on the gastrointestinal symptoms in patients with influenza report prevalence rates ranging from 0.6 to 6.6% for influenza A(H1N1) infections, and 9.8 to 57.5% for influenza A(H1N1)pdm09 infections, suggesting a possible association of gastrointestinal symptoms in our study with the 2009 swine flu [24].

Use of signs and symptoms of disease to rule out acute Q fever would be most appropriate in patients with non-severe lower respiratory tract infections, i.e., in cases where pneumonia is not suspected clinically. In such cases, use of antibiotics has been shown to provide little benefit in primary care, both overall and in patients aged 60 years and above, but may cause slight harms [25, 26]. Nevertheless, inappropriate use of antibiotics remains common in this population, as a study performed in 14,987 outpatients was recently able to show [27]. In a subgroup of 3306 patients with laboratory-confirmed influenza, in whom no pneumonia had been diagnosed, 945 (29%) were prescribed an antibiotic. Given the low yield of Q fever positives in wave 2 of our study, we assume that Q fever testing during wave 2 was in large part instigated by patients presenting with unspecific, but most likely influenza-related, symptoms. Although we have no data on rates of antibiotic prescriptions in this group, the percentage of subjects receiving inappropriate empiric doxycycline or other antibiotics may have been even higher than in aforementioned study. Therefore, under circumstances where outbreaks of Q fever overlap with other respiratory conditions, symptom-based prediction may deliver the greatest gain in terms of reducing antibiotic overuse.

In cases with clinical suspicion of pneumonia, however, the benefit of antibiotics would outweigh potential harms. For instance, several national guidelines recommend doxycycline as a second or first line drug for empiric treatment of community-acquired pneumonia (CAP), where it is generally considered to be safe and effective [28–32]. In cases of lower respiratory tract infections where acute Q fever is included in the differential diagnosis and pneumonia is suspected, use of doxycycline would thus seem an appropriate choice in an outpatient setting. The combination of doxycycline and hydroxychloroquine should be considered in patients with known valvular heart disease to prevent evolution to Q fever endocarditis (but is not recommended in patients with increased risk of acute Q fever endocarditis as revealed by high IgG anticardiolipin levels included in routine testing in some countries) [4, 5, 33]. Local antimicrobial resistance patterns are an important consideration in the choice of empirical treatment. While doxycycline is generally considered to be highly effective against atypical pathogens, including *C. burnetii*, doxycycline resistance is becoming more common in *Streptococcus pneumoniae*, particularly in isolates with reduced penicillin susceptibility. Although overall frequency of doxycycline resistance in *S. pneumoniae* in 2004 was 24%, rates vary widely geographically and over time, ranging from 2% to more than 20%, and more than 60% in penicillin-resistant strains, potentially limiting the use of doxycycline for more severe pneumococcal infections [34–37].

In our study, the prevalence of pneumonia in subjects tested during wave 1 was 23% overall, but 36% in Q fever positive subjects, which is higher than the 27% rate found in Q fever positive patients from a large 26-year cohort of patients with Q fever from the French National Reference Center for Q fever [38]. Nevertheless, a huge majority of patients in our study had no suspicion of pneumonia and would have had potential benefit from symptom-based exclusion of Q fever.

Predictive values are greatly impacted by prevalence of the disease in the base population. Positive predictive values (PPV) tend to be low in situations where prevalence in the base population is low, as was the case in our study, where post-outbreak seroprevalence of prior exposure to *C. burnetii* in the base population was estimated a mere 2.9% [1]. With PPV ranging between 4.6 and 8.3%, mirrored by low areas under the Receiver Operator Curves, our models had no use as a diagnostic tool for acute Q fever.

Conversely, negative predictive values (NPV) tend to be high under circumstances of low disease prevalence. With NPV ranging between 91.7 to 99.5%, our models were able to rule out the presence of acute Q fever with a relatively high degree of confidence. Nevertheless, decisions favouring a delayed or non-prescribing approach should ideally be corroborated by information from patient history, including self-reported exposures to farms, farm animals and farm animal products, and other clinical findings supporting such approach. In other contexts, for example in a well-circumscribed population of patients with high-risk exposure to a known source, prevalence may be (much) higher, with resulting decline in NPV.

To the best of our knowledge, ours is the first study to use post-outbreak data to validate prediction models for acute Q fever derived from outbreak data, thus enhancing the generalisability and robustness of our findings. Moreover, our study is first to assess the predictive potential of signs and symptoms for the diagnosis of acute Q fever in a large population of subjects most of whom were primary care patients. Other studies attempting to predict Q fever by signs and symptoms, including a retrospective case-control study from the Netherlands, were performed in hospital settings. The Dutch study reported that clinical signs and symptoms were not helpful in differentiating adult hospital-referred patients with acute Q fever from a hospital-referred control group [13]. A second study aimed to predict Q fever in patients presenting with community-acquired pneumonia to the hospital. The only symptom independently associated with Q fever in this study was headache. The prognostic score derived from multivariable logistic regression included male sex, age 30–60 years, a low leukocyte count and a high C-reactive protein (CRP) level, along with headache, as predictors of Q fever pneumonia [12]. A third study attempted to predict acute Q fever in febrile patients from rural Kenya, based on parameters including a range of clinical signs and symptoms. The study identified acute lower respiratory infection, abdominal pain, diarrhoea and a history of fever lasting > 14 days as independent significant positive predictors of acute Q fever. A prediction score derived from a modelling approach similar to ours was reported to reliably identify acute Q fever in febrile patients with undifferentiated illness [11].

Our study had a number of limitations. Selection of subjects for inclusion in our study was based on laboratory Q fever testing outcomes rather than random sampling, with a potential for selection bias, e.g., due to variations in diagnostic strategies between individual physicians. Laboratory confirmed cases of acute Q fever and patients who were Q fever negative were both selected based on signs and symptoms leading to addition of Q fever in the differential diagnosis, possibly resulting in some weakening of the association under study. Our laboratory data were strictly limited to outcomes of Q fever testing, precluding us from assessing signs and symptoms in relation to possible alternative outcomes. As mentioned above, misclassification of positive laboratory results as acute Q fever infection cannot be entirely ruled out, since phase-II IgM antibodies to *C. burnetii*, which at the time of the outbreak were considered to be reliable markers of acute Q fever infection, have been shown to persist for longer periods in individual patients, thus complicating the differentiation between past Q fever infections and acute respiratory infections with different aetiologies [39].

Validation and testing of our models were performed on samples from the same base population, potentially compromising generalisability of our findings. The lack of external validation of our models, however, may have partly been offset by the fact that we performed validation against a holdout sample, i.e., data from the second wave of Q fever testing. Testing during the second wave took place in what may be described as an immediate post-outbreak transition period where Q fever was increasingly replaced by other aetiologies of clinical respiratory disease, thus distinguishing the population of individuals tested during the second wave from those included in the first wave. Splitting our first-wave dataset for internal validation may have resulted in loss of power, and may have contributed to discrepancies between our four models in terms of predictors included in each model. Nevertheless, all four models showed poor to moderate performance in terms of AUC, but performed equally well in terms of their negative predictive value, suggesting that signs and symptoms of disease may be useful for symptom-based exclusion of acute Q fever. Whereas the Youden index is a commonly used method for cut-point selection in ROC analysis, there are several other approaches, whose application may have led to different results [40].

## Conclusions

Our study suggests that signs and symptoms of disease, considered in combination with age, sex and active smoking habits, do not accurately predict Q fever. However, presence of cough and gastrointestinal symptoms may point to different, possibly viral respiratory aetiologies, and help rule out acute fever in the absence suspected pneumonia and fever. In these cases, physicians in primary care may favour a delayed or non-prescribing approach if no known risk factors for progression to persistent focalized (or chronic) Q fever (e.g., heart valve or vascular anomalies) are present. A history of exposure to farms, farm animals or farm animal products may increase the likelihood of acute Q fever. It should be noted that PCR testing, whose sensitivity may be enhanced by lyophilisation, may shorten diagnostic delay and support early decision-making [41]. We recommend further validation of our findings in different larger independent cohorts.

## Data Availability

The datasets used and/or analysed during the current study are available from the corresponding author on reasonable request.

## Abbreviations

AUC: Area under the curve
CAP: Community-acquired pneumonia
CRP: C-reactive protein
GP: General practitioner
NPV: Negative predictive value
PCR: Polymerase chain reaction
PHS: Public health service
PPV: Positive predictive value
ROC: Receiver operator characteristic
